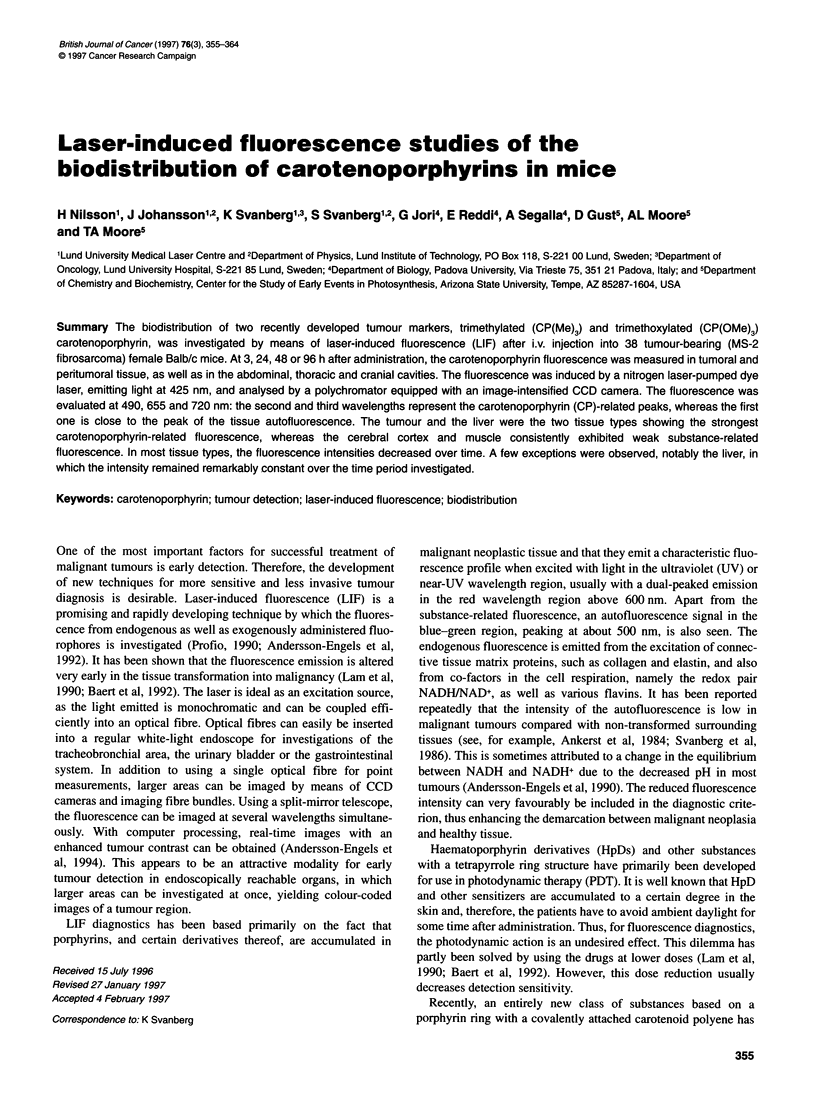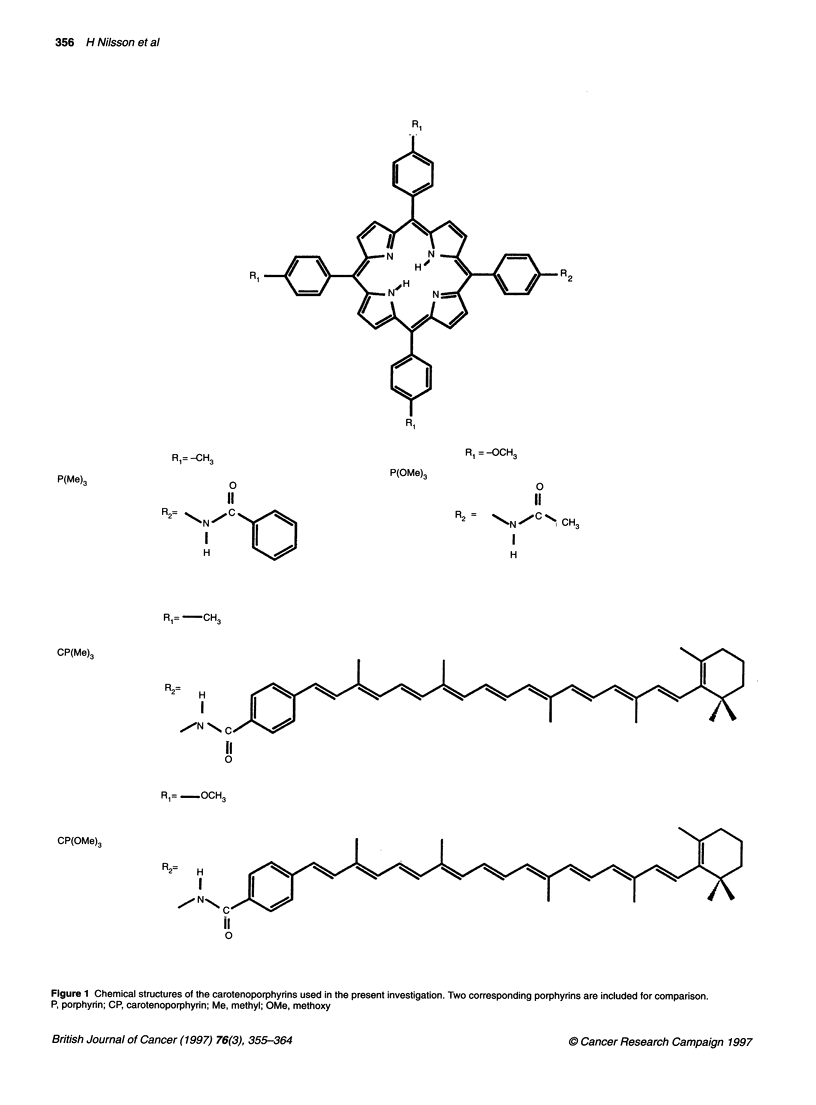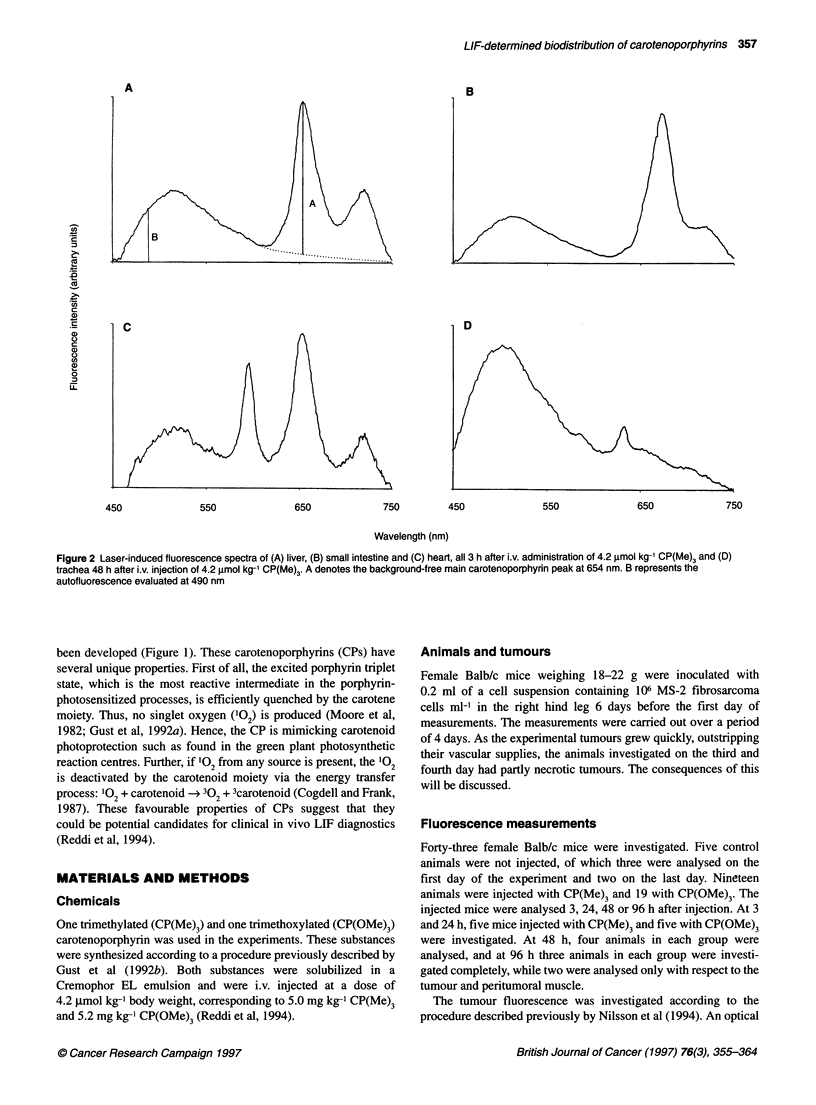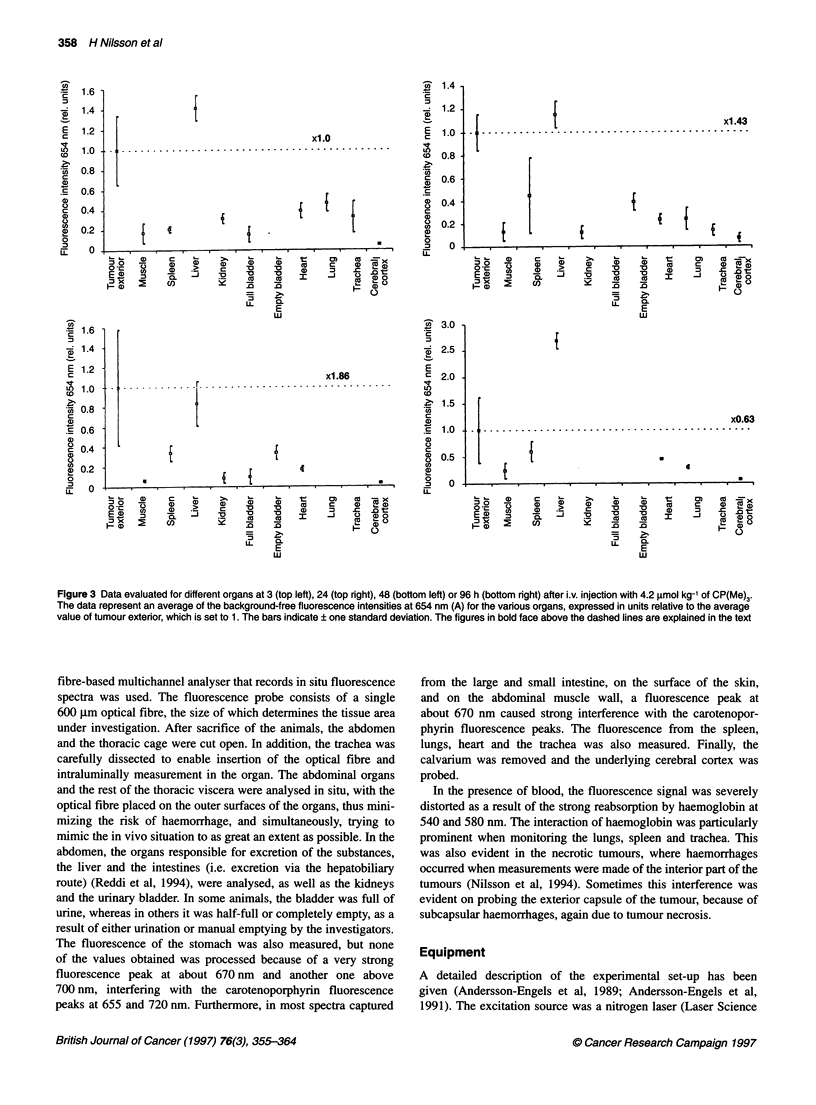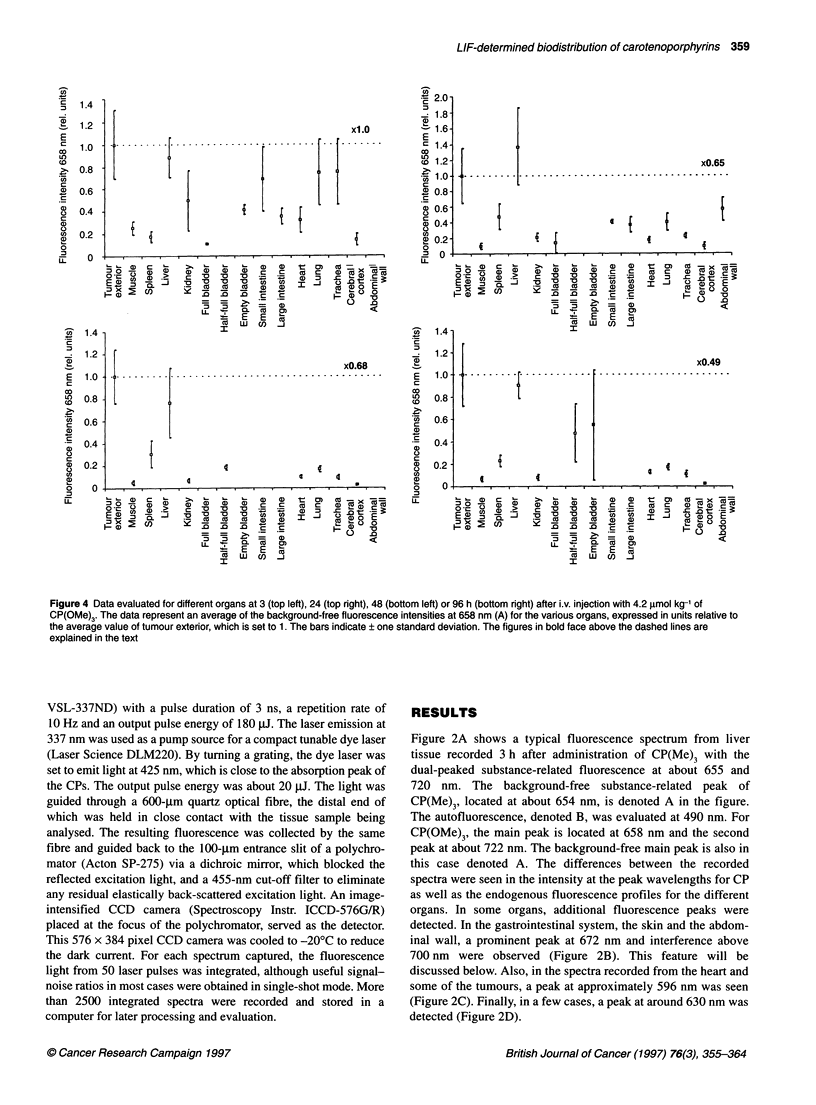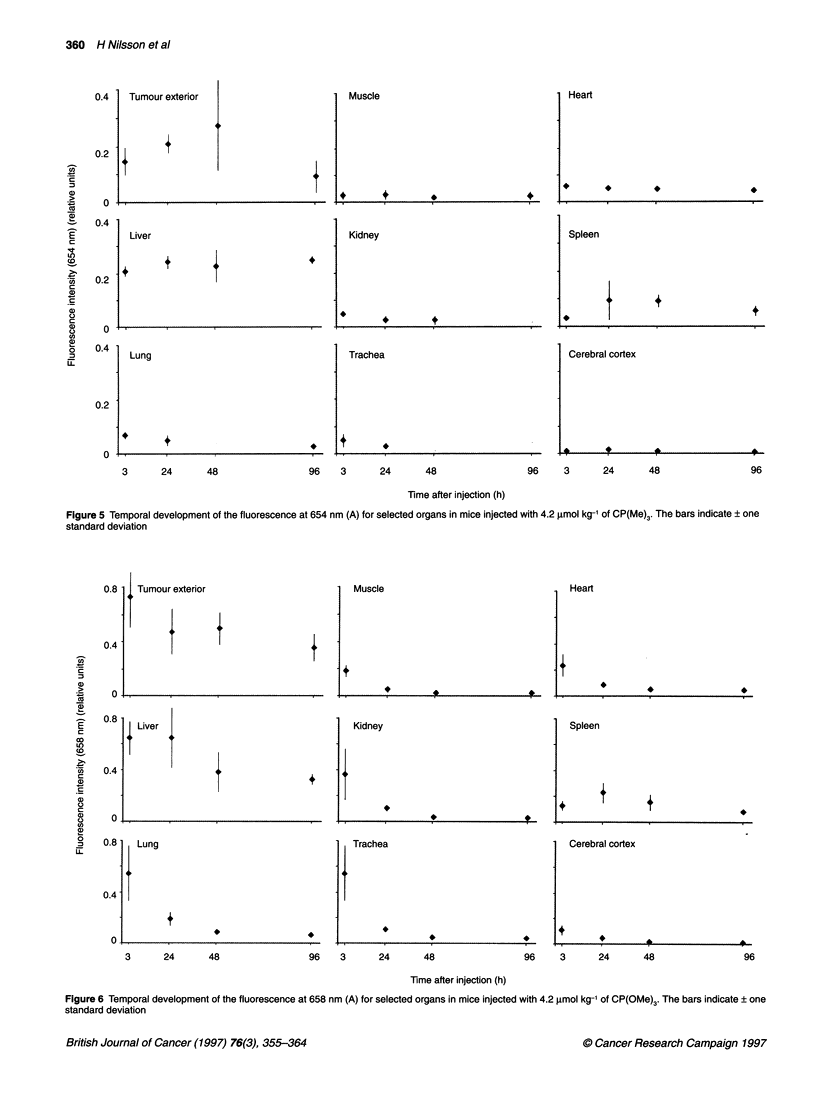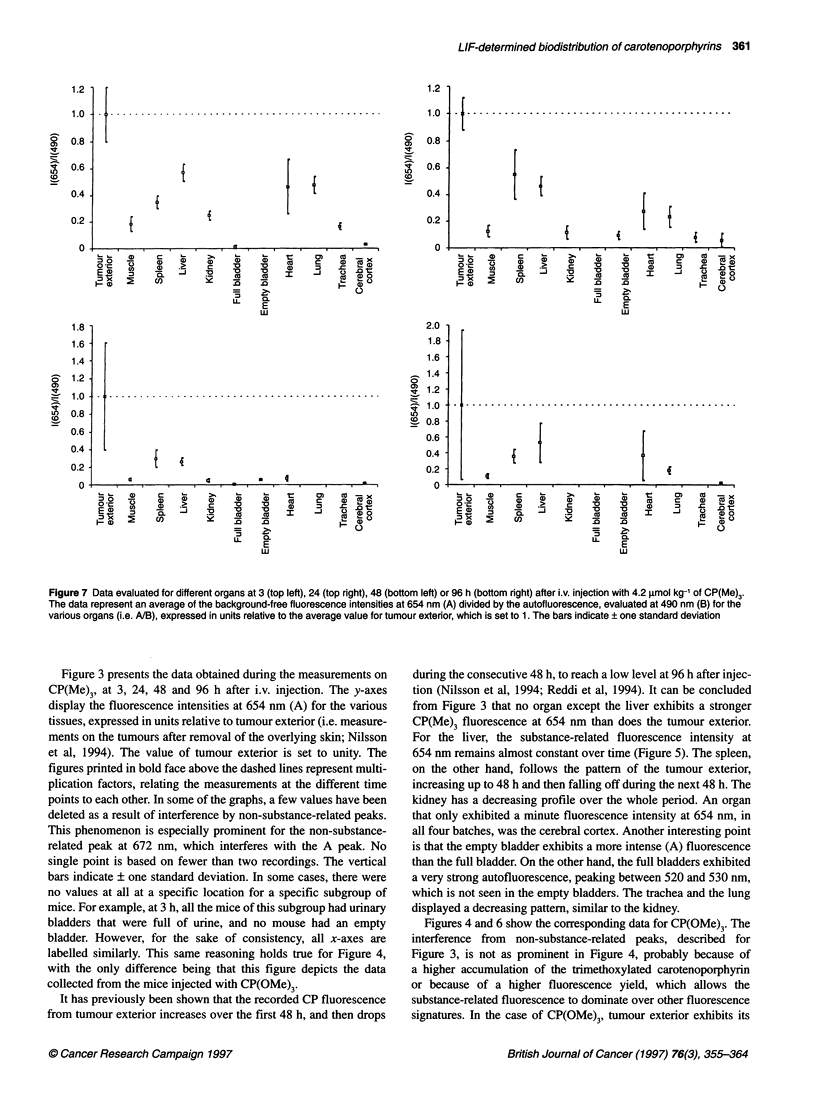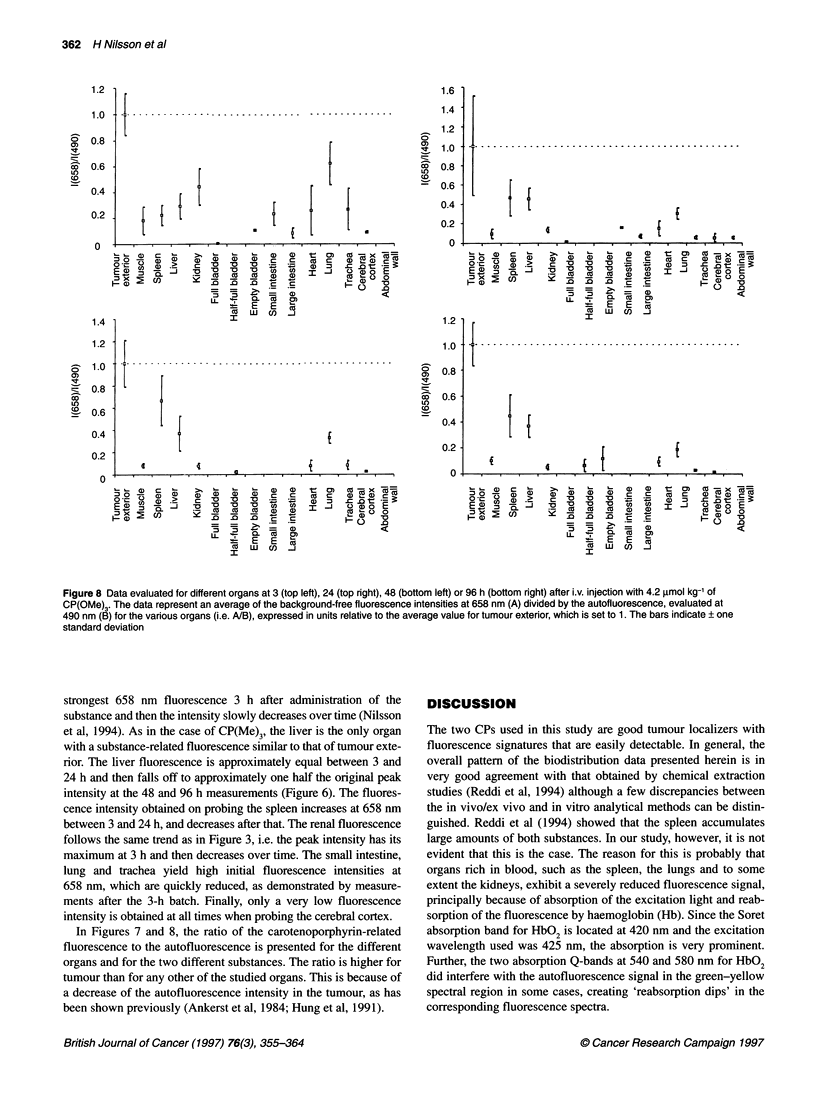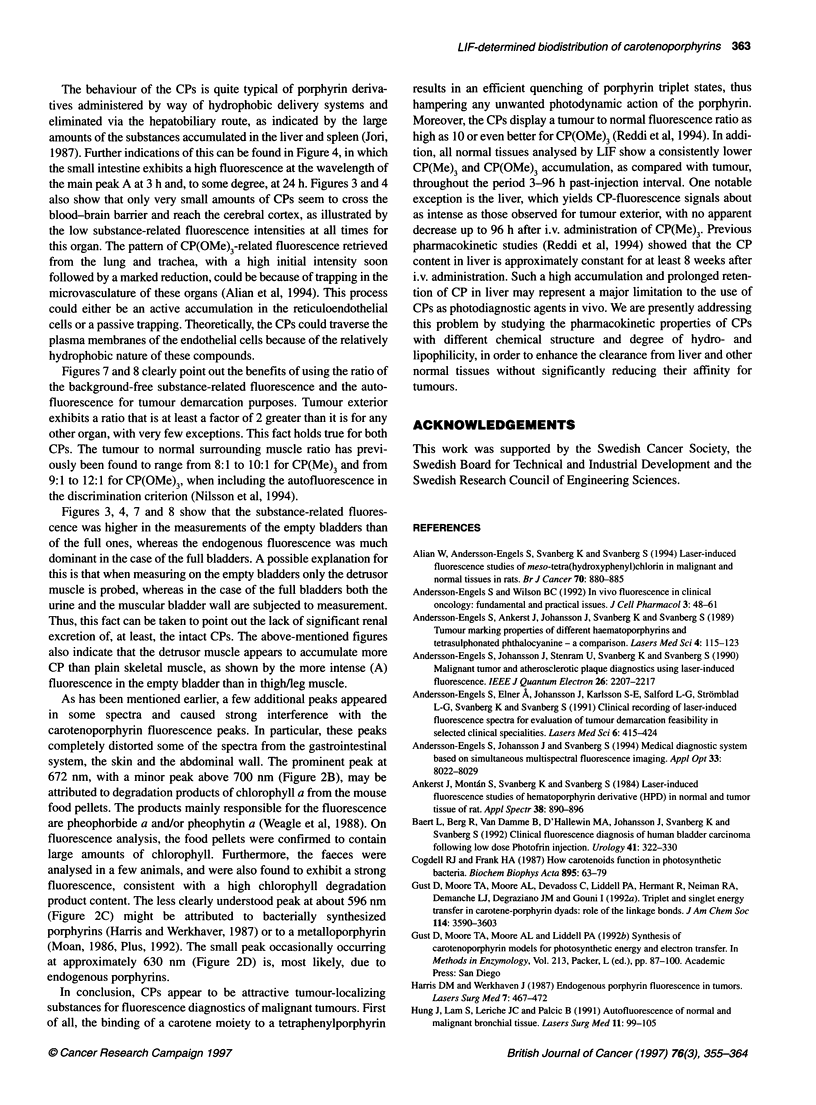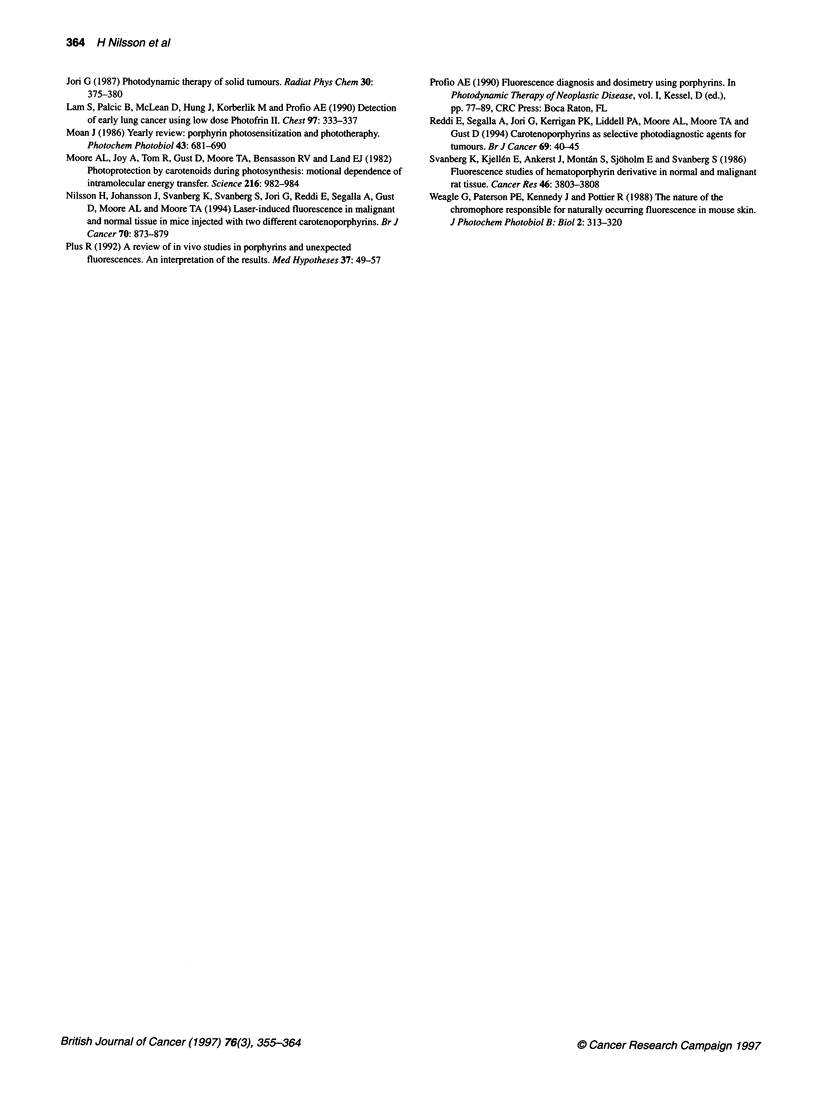# Laser-induced fluorescence studies of the biodistribution of carotenoporphyrins in mice.

**DOI:** 10.1038/bjc.1997.390

**Published:** 1997

**Authors:** H. Nilsson, J. Johansson, K. Svanberg, S. Svanberg, G. Jori, E. Reddi, A. Segalla, D. Gust, A. L. Moore, T. A. Moore

**Affiliations:** Lund University Medical Laser Centre, Lund Institute of Technology, Sweden.

## Abstract

The biodistribution of two recently developed tumour markers, trimethylated (CP(Me)3) and trimethoxylated (CP(OMe)3) carotenoporphyrin, was investigated by means of laser-induced fluorescence (LIF) after i.v. injection into 38 tumour-bearing (MS-2 fibrosarcoma) female Balb/c mice. At 3, 24, 48 or 96 h after administration, the carotenoporphyrin fluorescence was measured in tumoral and peritumoral tissue, as well as in the abdominal, thoracic and cranial cavities. The fluorescence was induced by a nitrogen laser-pumped dye laser, emitting light at 425 nm, and analysed by a polychromator equipped with an image-intensified CCD camera. The fluorescence was evaluated at 490, 655 and 720 nm: the second and third wavelengths represent the carotenoporphyrin (CP)-related peaks, whereas the first one is close to the peak of the tissue autofluorescence. The tumour and the liver were the two tissue types showing the strongest carotenoporphyrin-related fluorescence, whereas the cerebral cortex and muscle consistently exhibited weak substance-related fluorescence. In most tissue types, the fluorescence intensities decreased over time. A few exceptions were observed, notably the liver, in which the intensity remained remarkably constant over the time period investigated.


British Journal of Cancer (1997) 76(3), 355–364
© 1997 Cancer Research Campaign

### Laser-induced fluorescence studies of the biodistribution of carotenoporphyrins in mice

**H Nilsson[1], J Johansson[1,2], K Svanberg[1,3], S Svanberg[1,2], G Jori[4], E Reddi[4], A Segalla[4], D Gust[5], AL Moore[5] and TA Moore[5]**

[1]Lund University Medical Laser Centre and [2]Department of Physics, Lund Institute of Technology, PO Box 118, S-221 00 Lund, Sweden; [3]Department of Oncology, Lund University Hospital, S-221 85 Lund, Sweden; [4]Department of Biology, Padova University, Via Trieste 75, 351 21 Padova, Italy; and [5]Department of Chemistry and Biochemistry, Center for the Study of Early Events in Photosynthesis, Arizona State University, Tempe, AZ 85287-1604, USA

**Summary** The biodistribution of two recently developed tumour markers, trimethylated ($CP(Me)_3$) and trimethoxylated ($CP(OMe)_3$) carotenoporphyrin, was investigated by means of laser-induced fluorescence (LIF) after i.v. injection into 38 tumour-bearing (MS-2 fibrosarcoma) female Balb/c mice. At 3, 24, 48 or 96 h after administration, the carotenoporphyrin fluorescence was measured in tumoral and peritumoral tissue, as well as in the abdominal, thoracic and cranial cavities. The fluorescence was induced by a nitrogen laser-pumped dye laser, emitting light at 425 nm, and analysed by a polychromator equipped with an image-intensified CCD camera. The fluorescence was evaluated at 490, 655 and 720 nm: the second and third wavelengths represent the carotenoporphyrin (CP)-related peaks, whereas the first one is close to the peak of the tissue autofluorescence. The tumour and the liver were the two tissue types showing the strongest carotenoporphyrin-related fluorescence, whereas the cerebral cortex and muscle consistently exhibited weak substance-related fluorescence. In most tissue types, the fluorescence intensities decreased over time. A few exceptions were observed, notably the liver, in which the intensity remained remarkably constant over the time period investigated.

**Keywords:** carotenoporphyrin; tumour detection; laser-induced fluorescence; biodistribution

One of the most important factors for successful treatment of malignant tumours is early detection. Therefore, the development of new techniques for more sensitive and less invasive tumour diagnosis is desirable. Laser-induced fluorescence (LIF) is a promising and rapidly developing technique by which the fluorescence from endogenous as well as exogenously administered fluorophores is investigated (Profio, 1990; Andersson-Engels et al, 1992). It has been shown that the fluorescence emission is altered very early in the tissue transformation into malignancy (Lam et al, 1990; Baert et al, 1992). The laser is ideal as an excitation source, as the light emitted is monochromatic and can be coupled efficiently into an optical fibre. Optical fibres can easily be inserted into a regular white-light endoscope for investigations of the tracheobronchial area, the urinary bladder or the gastrointestinal system. In addition to using a single optical fibre for point measurements, larger areas can be imaged by means of CCD cameras and imaging fibre bundles. Using a split-mirror telescope, the fluorescence can be imaged at several wavelengths simultaneously. With computer processing, real-time images with an enhanced tumour contrast can be obtained (Andersson-Engels et al, 1994). This appears to be an attractive modality for early tumour detection in endoscopically reachable organs, in which larger areas can be investigated at once, yielding colour-coded images of a tumour region.

LIF diagnostics has been based primarily on the fact that porphyrins, and certain derivatives thereof, are accumulated in malignant neoplastic tissue and that they emit a characteristic fluorescence profile when excited with light in the ultraviolet (UV) or near-UV wavelength region, usually with a dual-peaked emission in the red wavelength region above 600 nm. Apart from the substance-related fluorescence, an autofluorescence signal in the blue–green region, peaking at about 500 nm, is also seen. The endogenous fluorescence is emitted from the excitation of connective tissue matrix proteins, such as collagen and elastin, and also from co-factors in the cell respiration, namely the redox pair $NADH/NAD^+$, as well as various flavins. It has been reported repeatedly that the intensity of the autofluorescence is low in malignant tumours compared with non-transformed surrounding tissues (see, for example, Ankerst et al, 1984; Svanberg et al, 1986). This is sometimes attributed to a change in the equilibrium between NADH and $NADH^+$ due to the decreased pH in most tumours (Andersson-Engels et al, 1990). The reduced fluorescence intensity can very favourably be included in the diagnostic criterion, thus enhancing the demarcation between malignant neoplasia and healthy tissue.

Haematoporphyrin derivatives (HpDs) and other substances with a tetrapyrrole ring structure have primarily been developed for use in photodynamic therapy (PDT). It is well known that HpD and other sensitizers are accumulated to a certain degree in the skin and, therefore, the patients have to avoid ambient daylight for some time after administration. Thus, for fluorescence diagnostics, the photodynamic action is an undesired effect. This dilemma has partly been solved by using the drugs at lower doses (Lam et al, 1990; Baert et al, 1992). However, this dose reduction usually decreases detection sensitivity.

Recently, an entirely new class of substances based on a porphyrin ring with a covalently attached carotenoid polyene has

*Received 15 July 1996*
*Revised 27 January 1997*
*Accepted 4 February 1997*

*Correspondence to:* K Svanberg

**Figure 1** Chemical structures of the carotenoporphyrins used in the present investigation. Two corresponding porphyrins are included for comparison. P, porphyrin; CP, carotenoporphyrin; Me, methyl; OMe, methoxy

© Cancer Research Campaign 1997

**Figure 2** Laser-induced fluorescence spectra of (A) liver, (B) small intestine and (C) heart, all 3 h after i.v. administration of 4.2 µmol kg$^{-1}$ $CP(Me)_3$ and (D) trachea 48 h after i.v. injection of 4.2 µmol kg$^{-1}$ $CP(Me)_3$. A denotes the background-free main carotenoporphyrin peak at 654 nm. B represents the autofluorescence evaluated at 490 nm been developed (Figure 1). These carotenoporphyrins (CPs) have several unique properties. First of all, the excited porphyrin triplet state, which is the most reactive intermediate in the porphyrin-photosensitized processes, is efficiently quenched by the carotene moiety. Thus, no singlet oxygen ($^1O_2$) is produced (Moore et al, 1982; Gust et al, 1992*a*). Hence, the CP is mimicking carotenoid photoprotection such as found in the green plant photosynthetic reaction centres. Further, if $^1O_2$ from any source is present, the $^1O_2$ is deactivated by the carotenoid moiety via the energy transfer process: $^1O_2$ + carotenoid → $^3O_2$ + $^3$carotenoid (Cogdell and Frank, 1987). These favourable properties of CPs suggest that they could be potential candidates for clinical in vivo LIF diagnostics (Reddi et al, 1994).

### MATERIALS AND METHODS

### Chemicals

One trimethylated ($CP(Me)_3$) and one trimethoxylated ($CP(OMe)_3$) carotenoporphyrin was used in the experiments. These substances were synthesized according to a procedure previously described by Gust et al (1992*b*). Both substances were solubilized in a Cremophor EL emulsion and were i.v. injected at a dose of 4.2 µmol kg$^{-1}$ body weight, corresponding to 5.0 mg kg$^{-1}$ $CP(Me)_3$ and 5.2 mg kg$^{-1}$ $CP(OMe)_3$ (Reddi et al, 1994).

### Animals and tumours

Female Balb/c mice weighing 18–22 g were inoculated with 0.2 ml of a cell suspension containing $10^6$ MS-2 fibrosarcoma cells ml$^{-1}$ in the right hind leg 6 days before the first day of measurements. The measurements were carried out over a period of 4 days. As the experimental tumours grew quickly, outstripping their vascular supplies, the animals investigated on the third and fourth day had partly necrotic tumours. The consequences of this will be discussed.

### Fluorescence measurements

Forty-three female Balb/c mice were investigated. Five control animals were not injected, of which three were analysed on the first day of the experiment and two on the last day. Nineteen animals were injected with $CP(Me)_3$ and 19 with $CP(OMe)_3$. The injected mice were analysed 3, 24, 48 or 96 h after injection. At 3 and 24 h, five mice injected with $CP(Me)_3$ and five with $CP(OMe)_3$ were investigated. At 48 h, four animals in each group were analysed, and at 96 h three animals in each group were investigated completely, while two were analysed only with respect to the tumour and peritumoral muscle.

The tumour fluorescence was investigated according to the procedure described previously by Nilsson et al (1994). An optical

**Figure 3** Data evaluated for different organs at 3 (top left), 24 (top right), 48 (bottom left) or 96 h (bottom right) after i.v. injection with 4.2 μmol $kg^{-1}$ of $CP(Me)_3$. The data represent an average of the background-free fluorescence intensities at 654 nm (A) for the various organs, expressed in units relative to the average value of tumour exterior, which is set to 1. The bars indicate ± one standard deviation. The figures in bold face above the dashed lines are explained in the text fibre-based multichannel analyser that records in situ fluorescence spectra was used. The fluorescence probe consists of a single 600 μm optical fibre, the size of which determines the tissue area under investigation. After sacrifice of the animals, the abdomen and the thoracic cage were cut open. In addition, the trachea was carefully dissected to enable insertion of the optical fibre and intraluminally measurement in the organ. The abdominal organs and the rest of the thoracic viscera were analysed in situ, with the optical fibre placed on the outer surfaces of the organs, thus minimizing the risk of haemorrhage, and simultaneously, trying to mimic the in vivo situation to as great an extent as possible. In the abdomen, the organs responsible for excretion of the substances, the liver and the intestines (i.e. excretion via the hepatobiliary route) (Reddi et al, 1994), were analysed, as well as the kidneys and the urinary bladder. In some animals, the bladder was full of urine, whereas in others it was half-full or completely empty, as a result of either urination or manual emptying by the investigators. The fluorescence of the stomach was also measured, but none of the values obtained was processed because of a very strong fluorescence peak at about 670 nm and another one above 700 nm, interfering with the carotenoporphyrin fluorescence peaks at 655 and 720 nm. Furthermore, in most spectra captured from the large and small intestine, on the surface of the skin, and on the abdominal muscle wall, a fluorescence peak at about 670 nm caused strong interference with the carotenoporphyrin fluorescence peaks. The fluorescence from the spleen, lungs, heart and the trachea was also measured. Finally, the calvarium was removed and the underlying cerebral cortex was probed.

In the presence of blood, the fluorescence signal was severely distorted as a result of the strong reabsorption by haemoglobin at 540 and 580 nm. The interaction of haemoglobin was particularly prominent when monitoring the lungs, spleen and trachea. This was also evident in the necrotic tumours, where haemorrhages occurred when measurements were made of the interior part of the tumours (Nilsson et al, 1994). Sometimes this interference was evident on probing the exterior capsule of the tumour, because of subcapsular haemorrhages, again due to tumour necrosis.

### Equipment

A detailed description of the experimental set-up has been given (Andersson-Engels et al, 1989; Andersson-Engels et al, 1991). The excitation source was a nitrogen laser (Laser Science

 © Cancer Research Campaign 1997

**Figure 4** Data evaluated for different organs at 3 (top left), 24 (top right), 48 (bottom left) or 96 h (bottom right) after i.v. injection with 4.2 μmol kg$^{-1}$ of $CP(OMe)_3$. The data represent an average of the background-free fluorescence intensities at 658 nm (A) for the various organs, expressed in units relative to the average value of tumour exterior, which is set to 1. The bars indicate ± one standard deviation. The figures in bold face above the dashed lines are explained in the text

VSL-337ND) with a pulse duration of 3 ns, a repetition rate of 10 Hz and an output pulse energy of 180 μJ. The laser emission at 337 nm was used as a pump source for a compact tunable dye laser (Laser Science DLM220). By turning a grating, the dye laser was set to emit light at 425 nm, which is close to the absorption peak of the CPs. The output pulse energy was about 20 μJ. The light was guided through a 600-μm quartz optical fibre, the distal end of which was held in close contact with the tissue sample being analysed. The resulting fluorescence was collected by the same fibre and guided back to the 100-μm entrance slit of a polychromator (Acton SP-275) via a dichroic mirror, which blocked the reflected excitation light, and a 455-nm cut-off filter to eliminate any residual elastically back-scattered excitation light. An image-intensified CCD camera (Spectroscopy Instr. ICCD-576G/R) placed at the focus of the polychromator, served as the detector. This 576 × 384 pixel CCD camera was cooled to –20°C to reduce the dark current. For each spectrum captured, the fluorescence light from 50 laser pulses was integrated, although useful signal–noise ratios in most cases were obtained in single-shot mode. More than 2500 integrated spectra were recorded and stored in a computer for later processing and evaluation.

### RESULTS

Figure 2A shows a typical fluorescence spectrum from liver tissue recorded 3 h after administration of $CP(Me)_3$ with the dual-peaked substance-related fluorescence at about 655 and 720 nm. The background-free substance-related peak of $CP(Me)_3$, located at about 654 nm, is denoted A in the figure. The autofluorescence, denoted B, was evaluated at 490 nm. For $CP(OMe)_3$, the main peak is located at 658 nm and the second peak at about 722 nm. The background-free main peak is also in this case denoted A. The differences between the recorded spectra were seen in the intensity at the peak wavelengths for CP as well as the endogenous fluorescence profiles for the different organs. In some organs, additional fluorescence peaks were detected. In the gastrointestinal system, the skin and the abdominal wall, a prominent peak at 672 nm and interference above 700 nm were observed (Figure 2B). This feature will be discussed below. Also, in the spectra recorded from the heart and some of the tumours, a peak at approximately 596 nm was seen (Figure 2C). Finally, in a few cases, a peak at around 630 nm was detected (Figure 2D).

© Cancer Research Campaign 1997

**Figure 5** Temporal development of the fluorescence at 654 nm (A) for selected organs in mice injected with 4.2 µmol kg$^{-1}$ of $CP(Me)_3$. The bars indicate ± one standard deviation

**Figure 6** Temporal development of the fluorescence at 658 nm (A) for selected organs in mice injected with 4.2 µmol kg$^{-1}$ of $CP(OMe)_3$. The bars indicate ± one standard deviation

 © Cancer Research Campaign 1997

**Figure 7** Data evaluated for different organs at 3 (top left), 24 (top right), 48 (bottom left) or 96 h (bottom right) after i.v. injection with 4.2 μmol $kg^{-1}$ of $CP(Me)_3$. The data represent an average of the background-free fluorescence intensities at 654 nm (A) divided by the autofluorescence, evaluated at 490 nm (B) for the various organs (i.e. A/B), expressed in units relative to the average value for tumour exterior, which is set to 1. The bars indicate ± one standard deviation

Figure 3 presents the data obtained during the measurements on $CP(Me)_3$, at 3, 24, 48 and 96 h after i.v. injection. The *y*-axes display the fluorescence intensities at 654 nm (A) for the various tissues, expressed in units relative to tumour exterior (i.e. measurements on the tumours after removal of the overlying skin; Nilsson et al, 1994). The value of tumour exterior is set to unity. The figures printed in bold face above the dashed lines represent multiplication factors, relating the measurements at the different time points to each other. In some of the graphs, a few values have been deleted as a result of interference by non-substance-related peaks. This phenomenon is especially prominent for the non-substance-related peak at 672 nm, which interferes with the A peak. No single point is based on fewer than two recordings. The vertical bars indicate ± one standard deviation. In some cases, there were no values at all at a specific location for a specific subgroup of mice. For example, at 3 h, all the mice of this subgroup had urinary bladders that were full of urine, and no mouse had an empty bladder. However, for the sake of consistency, all *x*-axes are labelled similarly. This same reasoning holds true for Figure 4, with the only difference being that this figure depicts the data collected from the mice injected with $CP(OMe)_3$.

It has previously been shown that the recorded CP fluorescence from tumour exterior increases over the first 48 h, and then drops during the consecutive 48 h, to reach a low level at 96 h after injection (Nilsson et al, 1994; Reddi et al, 1994). It can be concluded from Figure 3 that no organ except the liver exhibits a stronger $CP(Me)_3$ fluorescence at 654 nm than does the tumour exterior. For the liver, the substance-related fluorescence intensity at 654 nm remains almost constant over time (Figure 5). The spleen, on the other hand, follows the pattern of the tumour exterior, increasing up to 48 h and then falling off during the next 48 h. The kidney has a decreasing profile over the whole period. An organ that only exhibited a minute fluorescence intensity at 654 nm, in all four batches, was the cerebral cortex. Another interesting point is that the empty bladder exhibits a more intense (A) fluorescence than the full bladder. On the other hand, the full bladders exhibited a very strong autofluorescence, peaking between 520 and 530 nm, which is not seen in the empty bladders. The trachea and the lung displayed a decreasing pattern, similar to the kidney.

Figures 4 and 6 show the corresponding data for $CP(OMe)_3$. The interference from non-substance-related peaks, described for Figure 3, is not as prominent in Figure 4, probably because of a higher accumulation of the trimethoxylated carotenoporphyrin or because of a higher fluorescence yield, which allows the substance-related fluorescence to dominate over other fluorescence signatures. In the case of $CP(OMe)_3$, tumour exterior exhibits its

© *Cancer Research Campaign 1997*

**Figure 8** Data evaluated for different organs at 3 (top left), 24 (top right), 48 (bottom left) or 96 h (bottom right) after i.v. injection with 4.2 μmol $kg^{-1}$ of $CP(OMe)_3$. The data represent an average of the background-free fluorescence intensities at 658 nm (A) divided by the autofluorescence, evaluated at 490 nm (B) for the various organs (i.e. A/B), expressed in units relative to the average value for tumour exterior, which is set to 1. The bars indicate ± one standard deviation strongest 658 nm fluorescence 3 h after administration of the substance and then the intensity slowly decreases over time (Nilsson et al, 1994). As in the case of $CP(Me)_3$, the liver is the only organ with a substance-related fluorescence similar to that of tumour exterior. The liver fluorescence is approximately equal between 3 and 24 h and then falls off to approximately one half the original peak intensity at the 48 and 96 h measurements (Figure 6). The fluorescence intensity obtained on probing the spleen increases at 658 nm between 3 and 24 h, and decreases after that. The renal fluorescence follows the same trend as in Figure 3, i.e. the peak intensity has its maximum at 3 h and then decreases over time. The small intestine, lung and trachea yield high initial fluorescence intensities at 658 nm, which are quickly reduced, as demonstrated by measurements after the 3-h batch. Finally, only a very low fluorescence intensity is obtained at all times when probing the cerebral cortex.

In Figures 7 and 8, the ratio of the carotenoporphyrin-related fluorescence to the autofluorescence is presented for the different organs and for the two different substances. The ratio is higher for tumour than for any other of the studied organs. This is because of a decrease of the autofluorescence intensity in the tumour, as has been shown previously (Ankerst et al, 1984; Hung et al, 1991).

### DISCUSSION

The two CPs used in this study are good tumour localizers with fluorescence signatures that are easily detectable. In general, the overall pattern of the biodistribution data presented herein is in very good agreement with that obtained by chemical extraction studies (Reddi et al, 1994) although a few discrepancies between the in vivo/ex vivo and in vitro analytical methods can be distinguished. Reddi et al (1994) showed that the spleen accumulates large amounts of both substances. In our study, however, it is not evident that this is the case. The reason for this is probably that organs rich in blood, such as the spleen, the lungs and to some extent the kidneys, exhibit a severely reduced fluorescence signal, principally because of absorption of the excitation light and reabsorption of the fluorescence by haemoglobin (Hb). Since the Soret absorption band for $HbO_2$ is located at 420 nm and the excitation wavelength used was 425 nm, the absorption is very prominent. Further, the two absorption Q-bands at 540 and 580 nm for $HbO_2$ did interfere with the autofluorescence signal in the green–yellow spectral region in some cases, creating 'reabsorption dips' in the corresponding fluorescence spectra.

© Cancer Research Campaign 1997

The behaviour of the CPs is quite typical of porphyrin derivatives administered by way of hydrophobic delivery systems and eliminated via the hepatobiliary route, as indicated by the large amounts of the substances accumulated in the liver and spleen (Jori, 1987). Further indications of this can be found in Figure 4, in which the small intestine exhibits a high fluorescence at the wavelength of the main peak A at 3 h and, to some degree, at 24 h. Figures 3 and 4 also show that only very small amounts of CPs seem to cross the blood–brain barrier and reach the cerebral cortex, as illustrated by the low substance-related fluorescence intensities at all times for this organ. The pattern of $CP(OMe)_3$-related fluorescence retrieved from the lung and trachea, with a high initial intensity soon followed by a marked reduction, could be because of trapping in the microvasculature of these organs (Alian et al, 1994). This process could either be an active accumulation in the reticuloendothelial cells or a passive trapping. Theoretically, the CPs could traverse the plasma membranes of the endothelial cells because of the relatively hydrophobic nature of these compounds.

Figures 7 and 8 clearly point out the benefits of using the ratio of the background-free substance-related fluorescence and the autofluorescence for tumour demarcation purposes. Tumour exterior exhibits a ratio that is at least a factor of 2 greater than it is for any other organ, with very few exceptions. This fact holds true for both CPs. The tumour to normal surrounding muscle ratio has previously been found to range from 8:1 to 10:1 for $CP(Me)_3$ and from 9:1 to 12:1 for $CP(OMe)_3$, when including the autofluorescence in the discrimination criterion (Nilsson et al, 1994).

Figures 3, 4, 7 and 8 show that the substance-related fluorescence was higher in the measurements of the empty bladders than of the full ones, whereas the endogenous fluorescence was much dominant in the case of the full bladders. A possible explanation for this is that when measuring on the empty bladders only the detrusor muscle is probed, whereas in the case of the full bladders both the urine and the muscular bladder wall are subjected to measurement. Thus, this fact can be taken to point out the lack of significant renal excretion of, at least, the intact CPs. The above-mentioned figures also indicate that the detrusor muscle appears to accumulate more CP than plain skeletal muscle, as shown by the more intense (A) fluorescence in the empty bladder than in thigh/leg muscle.

As has been mentioned earlier, a few additional peaks appeared in some spectra and caused strong interference with the carotenoporphyrin fluorescence peaks. In particular, these peaks completely distorted some of the spectra from the gastrointestinal system, the skin and the abdominal wall. The prominent peak at 672 nm, with a minor peak above 700 nm (Figure 2B), may be attributed to degradation products of chlorophyll *a* from the mouse food pellets. The products mainly responsible for the fluorescence are pheophorbide *a* and/or pheophytin *a* (Weagle et al, 1988). On fluorescence analysis, the food pellets were confirmed to contain large amounts of chlorophyll. Furthermore, the faeces were analysed in a few animals, and were also found to exhibit a strong fluorescence, consistent with a high chlorophyll degradation product content. The less clearly understood peak at about 596 nm (Figure 2C) might be attributed to bacterially synthesized porphyrins (Harris and Werkhaver, 1987) or to a metalloporphyrin (Moan, 1986, Plus, 1992). The small peak occasionally occurring at approximately 630 nm (Figure 2D) is, most likely, due to endogenous porphyrins.

In conclusion, CPs appear to be attractive tumour-localizing substances for fluorescence diagnostics of malignant tumours. First of all, the binding of a carotene moiety to a tetraphenylporphyrin results in an efficient quenching of porphyrin triplet states, thus hampering any unwanted photodynamic action of the porphyrin. Moreover, the CPs display a tumour to normal fluorescence ratio as high as 10 or even better for $CP(OMe)_3$ (Reddi et al, 1994). In addition, all normal tissues analysed by LIF show a consistently lower $CP(Me)_3$ and $CP(OMe)_3$ accumulation, as compared with tumour, throughout the period 3–96 h past-injection interval. One notable exception is the liver, which yields CP-fluorescence signals about as intense as those observed for tumour exterior, with no apparent decrease up to 96 h after i.v. administration of $CP(Me)_3$. Previous pharmacokinetic studies (Reddi et al, 1994) showed that the CP content in liver is approximately constant for at least 8 weeks after i.v. administration. Such a high accumulation and prolonged retention of CP in liver may represent a major limitation to the use of CPs as photodiagnostic agents in vivo. We are presently addressing this problem by studying the pharmacokinetic properties of CPs with different chemical structure and degree of hydro- and lipophilicity, in order to enhance the clearance from liver and other normal tissues without significantly reducing their affinity for tumours.

### ACKNOWLEDGEMENTS

This work was supported by the Swedish Cancer Society, the Swedish Board for Technical and Industrial Development and the Swedish Research Council of Engineering Sciences.

### REFERENCES

Alian W, Andersson-Engels S, Svanberg K and Svanberg S (1994) Laser-induced fluorescence studies of *meso*-tetra(hydroxyphenyl)chlorin in malignant and normal tissues in rats. *Br J Cancer* **70**: 880–885

Andersson-Engels S and Wilson BC (1992) In vivo fluorescence in clinical oncology: fundamental and practical issues. *J Cell Pharmacol* **3**: 48–61

Andersson-Engels S, Ankerst J, Johansson J, Svanberg K and Svanberg S (1989) Tumour marking properties of different haematoporphyrins and tetrasulphonated phthalocyanine – a comparison. *Lasers Med Sci* **4**: 115–123

Andersson-Engels S, Johansson J, Stenram U, Svanberg K and Svanberg S (1990) Malignant tumor and atherosclerotic plaque diagnostics using laser-induced fluorescence. *IEEE J Quantum Electron* **26**: 2207–2217

Andersson-Engels S, Elner Å, Johansson J, Karlsson S-E, Salford L-G, Strömblad L-G, Svanberg K and Svanberg S (1991) Clinical recording of laser-induced fluorescence spectra for evaluation of tumour demarcation feasibility in selected clinical specialities. *Lasers Med Sci* **6**: 415–424

Andersson-Engels S, Johansson J and Svanberg S (1994) Medical diagnostic system based on simultaneous multispectral fluorescence imaging. *Appl Opt* **33**: 8022–8029

Ankerst J, Montán S, Svanberg K and Svanberg S (1984) Laser-induced fluorescence studies of hematoporphyrin derivative (HPD) in normal and tumor tissue of rat. *Appl Spectr* **38**: 890–896

Baert L, Berg R, Van Damme B, D'Hallewin MA, Johansson J, Svanberg K and Svanberg S (1992) Clinical fluorescence diagnosis of human bladder carcinoma following low dose Photofrin injection. *Urology* **41**: 322–330

Cogdell RJ and Frank HA (1987) How carotenoids function in photosynthetic bacteria. *Biochem Biophys Acta* **895**: 63–79

Gust D, Moore TA, Moore AL, Devadoss C, Liddell PA, Hermant R, Neiman RA, Demanche LJ, Degraziano JM and Gouni I (1992*a*). Triplet and singlet energy transfer in carotene-porphyrin dyads: role of the linkage bonds. *J Am Chem Soc* **114**: 3590–3603

Gust D, Moore TA, Moore AL and Liddell PA (1992*b*) Synthesis of carotenoporphyrin models for photosynthetic energy and electron transfer. In *Methods in Enzymology*, Vol. 213, Packer, L (ed.), pp. 87–100. Academic Press: San Diego

Harris DM and Werkhaven J (1987) Endogenous porphyrin fluorescence in tumors. *Lasers Surg Med* **7**: 467–472

Hung J, Lam S, Leriche JC and Palcic B (1991) Autofluorescence of normal and malignant bronchial tissue. *Lasers Surg Med* **11**: 99–105

© Cancer Research Campaign 1997

Jori G (1987) Photodynamic therapy of solid tumours. *Radiat Phys Chem* **30**: 375–380

Lam S, Palcic B, McLean D, Hung J, Korberlik M and Profio AE (1990) Detection of early lung cancer using low dose Photofrin II. *Chest* **97**: 333–337

Moan J (1986) Yearly review: porphyrin photosensitization and phototheraphy. *Photochem Photobiol* **43**: 681–690

Moore AL, Joy A, Tom R, Gust D, Moore TA, Bensasson RV and Land EJ (1982) Photoprotection by carotenoids during photosynthesis: motional dependence of intramolecular energy transfer. *Science* **216**: 982–984

Nilsson H, Johansson J, Svanberg K, Svanberg S, Jori G, Reddi E, Segalla A, Gust D, Moore AL and Moore TA (1994) Laser-induced fluorescence in malignant and normal tissue in mice injected with two different carotenoporphyrins. *Br J Cancer* **70**: 873–879

Plus R (1992) A review of in vivo studies in porphyrins and unexpected fluorescences. An interpretation of the results. *Med Hypotheses* **37**: 49–57

Profio AE (1990) Fluorescence diagnosis and dosimetry using porphyrins. In *Photodynamic Therapy of Neoplastic Disease*, vol. I, Kessel, D (ed.), pp. 77–89, CRC Press: Boca Raton, FL

Reddi E, Segalla A, Jori G, Kerrigan PK, Liddell PA, Moore AL, Moore TA and Gust D (1994) Carotenoporphyrins as selective photodiagnostic agents for tumours. *Br J Cancer* **69**: 40–45

Svanberg K, Kjellén E, Ankerst J, Montán S, Sjöholm E and Svanberg S (1986) Fluorescence studies of hematoporphyrin derivative in normal and malignant rat tissue. *Cancer Res* **46**: 3803–3808

Weagle G, Paterson PE, Kennedy J and Pottier R (1988) The nature of the chromophore responsible for naturally occurring fluorescence in mouse skin. *J Photochem Photobiol B: Biol* **2**: 313–320

© Cancer Research Campaign 1997